# A mechanistic stress model of protein evolution accounts for site-specific evolutionary rates and their relationship with packing density and flexibility

**DOI:** 10.1186/1471-2148-14-78

**Published:** 2014-04-09

**Authors:** Tsun-Tsao Huang, María Laura del Valle Marcos, Jenn-Kang Hwang, Julian Echave

**Affiliations:** 1 Institute of Bioinformatics and Systems Biology, National Chiao Tung University, HsinChu 30050, Taiwan; 2 Center for Bioinformatics Research, National Chiao Tung University, HsinChu 30050, Taiwan; 3 Escuela de Ciencia y Tecnología, Universidad Nacional de San Martín, Martín de Irigoyen 3100, 1650 San Martín, Buenos Aires Argentina

**Keywords:** Protein evolution, Site-specific substitution rate, Local packing density, Elastic network model, Flexibility, Stress, Mean square fluctuation, Mean local mutational stress

## Abstract

**Background:**

Protein sites evolve at different rates due to functional and biophysical constraints. It is usually considered that the main structural determinant of a site’s rate of evolution is its Relative Solvent Accessibility (RSA). However, a recent comparative study has shown that the main structural determinant is the site’s Local Packing Density (LPD). LPD is related with dynamical flexibility, which has also been shown to correlate with sequence variability. Our purpose is to investigate the mechanism that connects a site’s LPD with its rate of evolution.

**Results:**

We consider two models: an empirical Flexibility Model and a mechanistic Stress Model. The Flexibility Model postulates a linear increase of site-specific rate of evolution with dynamical flexibility. The Stress Model, introduced here, models mutations as random perturbations of the protein’s potential energy landscape, for which we use simple Elastic Network Models (ENMs). To account for natural selection we assume a single active conformation and use basic statistical physics to derive a linear relationship between site-specific evolutionary rates and the local stress of the mutant’s active conformation.

We compare both models on a large and diverse dataset of enzymes. In a protein-by-protein study we found that the Stress Model outperforms the Flexibility Model for most proteins. Pooling all proteins together we show that the Stress Model is strongly supported by the total weight of evidence. Moreover, it accounts for the observed nonlinear dependence of sequence variability on flexibility. Finally, when mutational stress is controlled for, there is very little remaining correlation between sequence variability and dynamical flexibility.

**Conclusions:**

We developed a mechanistic Stress Model of evolution according to which the rate of evolution of a site is predicted to depend linearly on the local mutational stress of the active conformation. Such local stress is proportional to LPD, so that this model explains the relationship between LPD and evolutionary rate. Moreover, the model also accounts for the nonlinear dependence between evolutionary rate and dynamical flexibility.

## Background

Due to functional and biophysical constraints, different protein sites evolve at different rates of amino-acid substitution [1-6]. The most popular structural correlate of a site’s substitution rate is its Relative Solvent Accessibility (RSA) [7-10]. In a thorough assessment of many structural properties as predictors of site-specific rates of evolution, Franzosa and Xia showed that the only two with significant independent contributions are RSA and CN, the Contact Number, with RSA performing slightly better [9]. However, in a more recent study, Yeh et al. compared RSA with two Local Packing Density (LPD) measures, CN and the Weighted Contact Number (WCN), and found that both LPD measures correlate better than RSA with evolutionary rates [11]. Moreover, they found that once LPD is controlled for, the independent contribution of RSA is small. Thus, LPD seems to be the main structural determinant of rate of evolution at site level. The purpose of the present work is to study possible mechanisms that connect LPD to evolutionary rates.

A possible link could be dynamical flexibility. A site’s flexibility, quantified by its Mean Squared Fluctuation (MSF), is approximately proportional to 1/LPD [12]. A flexibility-based explanation assumes that a site’s rate of evolution increases with its dynamical flexibility. Within this framework 1/LPD would be just a “proxy” of a site’s flexibility, which would be the actual determinant of its evolutionary rate. Such interpretation would seem to be supported by empirical correlation studies of sequence variability vs. MSF [13] and variability vs. 1/LPD [14,15], and by a recent study based on a different dynamical flexibility measure [16]. Such a flexibility-based explanation not only makes some intuitive sense, but it is attractive because it is in line with the increasing acknowledgement of the role of dynamics for protein function [17,18]. Therefore, we postulate as our null model an explicit empirical Flexibility Model according to which a site’s rate of evolution depends linearly on its MSF.

The main drawback of the previous flexibility-based interpretations, and the empirical Flexibility Model we set up to make their underlying assumptions explicit, is that no mechanism is proposed. To this end, here we propose a mechanistic alternative model. We model mutations as random perturbations of the parameters of the protein’s potential energy landscape and natural selection as a function of the probability that a mutant adopts a specific active conformation. Using basic statistical physics and certain simplifying assumptions, we derive that according to this model a site’s evolutionary rate will depend on the local stress introduced in the active structure by mutating it. Therefore, we shall call it the Stress Model.

We will show that the Stress Model explains both the dependence of site-specific rates of evolution on packing density and on dynamical flexibility in terms of the local stress introduced by mutations on the protein’s active structure.

## Methods

### Elastic network models

Let the conformation of an N-sites protein be represented by the column vector of the 3 N Cartesian coordinates of its N *C*_*α*_ atoms: ***r*** = (*x*_1_ *y*_1_ *z*_1_ *x*_2_ *y*_2_ *z*_2_ … *x*_*N*_ *y*_*N*_ *z*_*N*_ )^*T*^. ***r***_***i***_ = (*x*_*i*_ *y*_*i*_ *z*_*i*_)^*T*^ is the position vector of the *i*th *C*_*α*_. The vector joining sites *i*  and *j* is ***d***_*ij*_ = ***r***_*j*_ - ***r***_*i*_ with length *d*_*ij*_ = ***d***_*ij*_. We use ***r***^0^ for the protein’s equilibrium conformation in which the *i*th site is at $r_{i}^{0}$.

An Elastic Network Model (ENM) represents the folded protein as a network of sites connected by springs. They have proved accurate and useful in a variety of applications [17,19]. The potential energy landscape is given by:

(1) $$V\left(r\right)=\frac{1}{2}\sum_{i=1}^{N-1}\sum_{j=i+1}^{N}k_{\mathrm{ij}}{\left(d_{\mathrm{ij}}-d_{\mathrm{ij}}^{0}\right)}^{2}$$

where $d_{\mathrm{ij}}^{0}$ and *k*_*ij*_ are, respectively, the equilibrium length and force constant of spring *ij*. As far as we know, all models proposed so far assume that $d_{\mathrm{ij}}^{0}=d_{\mathrm{ij}}\left(r^{0}\right)=r_{j}^{0}-r_{i}^{0}$, i.e. that at the equilibrium conformation ***r***^0^, all springs are relaxed.

### Fluctuations and flexibility

No protein is frozen at its equilibrium conformation. At non-zero absolute temperature, the folded protein fluctuates around  ***r***^0^ sampling conformational space with equilibrium Boltzmann’s probability density function:

(2) $$\rho \left(r\right)=\frac{e^{-\mathrm{\beta V}\left(r\right)}}{Z_{F}}$$

where β=1kBT, with *T* the absolute temperature and *k*_*B*_ Boltzmann’s constant. The denominator of Eq. (2) is the partition function of the folded protein:

(3) $$Z_{F}=\int e^{-\mathrm{\beta V}\left(r\right)}\mathrm{d\tau}$$

where ∫ … *dτ* stands for integration over the whole of conformational space.

The dynamical flexibility (mobility) of a site is ordinarily quantified using its Mean Square Fluctuation:

(4) $$\mathrm{MS}F_{i}\equiv \langle {║r_{i}-r_{i}^{0}║}^{2}\rangle =\int {║r_{i}-r_{i}^{0}║}^{2}\rho \left(r\right)\mathrm{d\tau}$$

To calculate *MSF*_*i*_ using Eq. (4), the potential energy function Eq. (1) is approximated using a second-order Taylor expansion around its equilibrium conformation. First, the Hessian matrix ***H*** of second derivatives of the potential Eq. (1) with respect to the atoms’ Cartesian coordinates is calculated. Then, ***H*** is inverted to obtain the 3*N* × 3*N* variance-covariance matrix ***C***, which is composed of a 3 × 3 ***C***_*ij*_ block for each pair of sites. Finally, a site’s MSF is given by [20]:

(5) $$\mathrm{MS}F_{i}=\mathrm{Tr}\left(C_{\mathrm{ii}}\right)$$

### An empirical flexibility model

Several studies have investigated the correlation between site-specific rates of evolution or other sequence-variability measures and the corresponding flexibility. Since such studies use Pearson’s correlation coefficients as measure of association, the underlying assumption is that there is a linear relationship between rate of evolution and flexibility. To make such assumption explicit, here we postulate the following Flexibility Model:

(6) $${\tilde{\omega_{i}}}^{\mathrm{FLEX}}=a_{P}^{\mathrm{FLEX}}+b_{P}^{\mathrm{FLEX}}\tilde{\mathrm{MS}F_{i}}$$

where $\tilde{\omega_{i}}\;$ is the relative rate of substitution of the *i*th site. In general, for site-specific scalar properties we will use relative values obtained by z-score normalization. For any given site-specific property *x*_*i*_, we the z-score normalized values are xi˜=xi-xx2-x2, where the averages are calculated over all sites of the same protein. The subscript *P* is used to note that *a priori* the coefficients may depend on the protein considered. We emphasize that the Flexibility Model is empirical: rather than derived from first principles, it is postulated, based on the intuitive notion that flexible sites should accommodate mutations more easily.

### A mechanistic stress model

We introduce here a mechanistic model that includes explicitly the effects of mutations and natural selection. We consider mutations as random perturbations of the wild-type ENM potential [21-23]. A random mutation at site *i* results in a mutant whose potential *V*_*mut*_ is obtained from Eq. (1) by adding perturbations to the equilibrium length of each of its springs: $d_{\mathrm{ij}}^{0}\to d_{\mathrm{ij}}^{0}+\delta_{\mathrm{ij}}.$ We further assume that the springs are independently perturbed and that perturbations are spring-independent, randomly drawn from a distribution with zero mean and constant variance *α*^2^:

(7) $$\langle \delta_{\mathrm{ij}}\rangle =0;\langle \delta_{\mathrm{ij}}^{2}\rangle =\alpha^{2}.$$

As we mentioned above, when the wild type is at its equilibrium conformation $r_{\mathrm{wt}}^{0}$, all springs are relaxed by construction. In contrast, when the mutant is at $r_{\mathrm{wt}}^{0}$, the mutated site’s springs will be stressed (stretched or compressed). For further reference, we define the Mean Local mutational Stress (MLmS) as follows:

(8) $$\mathrm{MLm}S_{i}\equiv {\langle V_{\mathrm{mut}}\left(r_{\mathrm{wt}}^{0}\right)-V_{\mathrm{wt}}\left(r_{\mathrm{wt}}^{0}\right)\rangle }_{\mathrm{mut}@i}$$

where 〈 … 〉_*mut* @ *i*_ stands for averaging over random mutations at the *i*th site.

To complete the model, we derive a simple selection function. First, we assume that there is a single specific active conformation ***r***_*active*_. Next, we acknowledge fluctuations and assume that the protein’s activity (either the wild-type’s or a mutant’s) is proportional to the concentration of the active conformation ***r***_*active*_. Finally, we assume that $r_{\mathrm{active}}=r_{\mathrm{wt}}^{0}$ and, accordingly, we model the acceptance probability of a mutant as:

(9) $$p^{\mathrm{accept}}\equiv \frac{C_{\mathrm{mut}}^{F}\rho_{\mathrm{mut}}\left(r_{\mathrm{wt}}^{0}\right)}{C_{\mathrm{wt}}^{F}\rho_{\mathrm{wt}}\left(r_{\mathrm{wt}}^{0}\right)}$$

Where $C_{\mathrm{mut}}^{F}$ and $C_{\mathrm{wt}}^{F}$ are the concentrations of folded protein for the mutant and wild type, respectively. From statistical mechanics, the Folded-Unfolded equilibrium constants for the wild-type and mutant proteins are, respectively, CwtFCwtU=ZwtFZwtU and CmutFCmutU=ZmutFZmutU. We further assume that the partition function and concentration of unfolded protein is the same for the mutant and wild type. Therefore CmutFCwtF=ZmutZwt. Replacing this relationship and Eq. (2) into Eq. (9) we find:

(10) $$p^{\mathrm{accept}}=e^{-\beta \;\left[V_{\mathrm{mut}}\left(r_{\mathrm{wt}}^{0}\right)-V_{\mathrm{wt}}\left(r_{\mathrm{wt}}^{0}\right)\right]}$$

Finally, averaging over random mutations at site *i* and using Eq. (8) we obtain the *acceptance rate*:

(11) $$\omega_{i}\equiv {\langle p_{i}^{\mathrm{accept}}\rangle }_{\mathrm{mut}@i}={\langle e^{-\beta \;\left[V_{\mathrm{mut}}\left(r_{\mathrm{wt}}^{0}\right)-V_{\mathrm{wt}}\left(r_{\mathrm{wt}}^{0}\right)\right]}\rangle }_{\mathrm{mut}@i}≅1-\beta \;\mathrm{MLm}S_{i}$$

Where *β* may be thought of as representing not just temperature but also selection pressure, and we have assumed that *β*Δ*V* << 1 (mild selection) to approximate the exponential to first order. To finish, we z-normalize the variables of Eq. (11) to get the relative rates of evolution:

(12) $${\tilde{\omega_{i}}}^{\mathrm{STRESS}}=a_{P}^{\mathrm{STRESS}}+b_{P}^{\mathrm{STRESS}}\tilde{\mathrm{MLm}S_{i}}.$$

This equation specifies the stress model.

### Relationship of flexibility and stress with packing density

The purpose of this work is to investigate why LPD correlates with rate of evolution at site level. The previous models relate rates of evolution with MSF (Eq. 6) and MLmS (Eq. 12). Here we derive the relationship between these properties and LPD measures.

First, we relate flexibility and stress with the potential energy parameters of Eq. (1). Let us define:

(13) $$k_{i}\equiv \sum_{j\neq i}k_{\mathrm{ij}}$$

Regarding flexibility, replacing Eqs. (1), (2), and (3) into Eq. (4), following [12], and using Eq. (13), it can be found that:

(14) $$\mathrm{MS}F_{i}≅\frac{3}{2\beta k_{i}}$$

Regarding stress, from Eqs. (1), (7), and (8), after some algebra, we get:

(15) $$\mathrm{MLm}S_{i}=\frac{1}{2}\alpha^{2}k_{i}$$

Note that Eq. (14) is an approximation while Eq. (15) is an identity.

Second, to relate the previous models to LPD we need to specify the ENMs spring constants. A variety of ENMs have been developed (see [24] for a recent comparison). Here, we consider two models. First, the “parameter-free Anisotropic Network Model” (pfANM) [25], which uses:

(16) $$k_{\mathrm{ij}}=\frac{1}{{\left(d_{\mathrm{ij}}^{0}\right)}^{2}}$$

Second, the “Anisotropic Network Model” (ANM) [20], for which:

(17) $$k_{\mathrm{ij}}=\left\{\begin{array}{cc} 1 & d_{\mathrm{ij}}^{0}\leq R_{\mathrm{cut}}\\ 0 & d_{\mathrm{ij}}^{0}>R_{\mathrm{cut}} \end{array}\right)$$

where *R*_*cut*_ is typically between 10 Å and 18 Å.

From Eqs. (13), (16), and (17) and z-normalizing we find:

(18) $$\tilde{k_{i}}=\tilde{\mathrm{LP}D_{i}}$$

where for the pfANM, LPD is the Weighted Contact Number (WCN) of [26], and for the ANM, it is the Contact Number (CN): the number of sites closer than *R*_*cut*_. Finally, from Eqs. (14) and (18) it follows:

(19) $$\tilde{\mathrm{MS}F_{i}}≅{\tilde{k_{i}}}^{-1}={\tilde{\mathrm{LP}D_{i}}}^{-1}$$

Similarly, from Eqs. (15) and (18) we get:

(20) $$\tilde{\mathrm{MLm}S_{i}}=\tilde{k_{i}}=\tilde{\mathrm{LP}D_{i}}$$

Note that while MSF is approximately equal to 1/LPD, MLmS is exactly equal to LPD (for relative z-normalized values).

### Calculation details

We used the dataset of 213 monomeric enzymes of Yeh et al. [11]. The dataset includes proteins of diverse sizes, functional, and structural classes (Additional file 1: Table S1).

We used the evolutionary rates of [11]. They were inferred from multiple alignments of homologous sequences using Rate4Site, which builds the phylogenetic tree using a neighbour-joining algorithm and estimates rates with an empirical Bayesian approach and the JTT model of sequence evolution [27,28]. To keep in mind that we are not dealing with the (unknown) “true rates”, but with Rate4Site-inferred rates, we use the notation ${\tilde{\omega }}_{i}^{R4S}$.

From the pdb equilibrium structure of each protein we calculated the spring constants of pfANM (Eq. 16) and ANM (Eq. 17), for which we used a cut-off distance of 13 Å [11]. Given a protein and ENM model, we calculated the Hessian matrix, inverted it to obtain the variance-covariance matrix, and calculated the site-specific flexibility values $\tilde{\mathrm{MS}F_{i}}$ using Eq. (5) and z-normalizing. Regarding stress, we obtained the relative site-specific values $\tilde{\mathrm{MLm}S_{i}}$ using Eq. (15) and z-normalizing.

Since we always use z-normalized relative values, for the sake of notational simplicity, we shall use *ω*^*R*4*S*^, *MSF*, and *MLmS* to refer to z-normalized values from now on.

We performed two analyses. In a protein-by-protein analysis, we performed linear fits of *ω*^*R*4*S*^ with either *MSF* (Flexibility Model) or *MLmS* (Stress Model) using the lm() function of the base package of R for each protein. In a global analysis we pooled together all sites of all proteins and performed similar global fits.

To assess the goodness-of-fit of a model to the data, we used the Akaike Information Criterion *AIC* = 2*k* - 2 ln *L*, where *k* is the number of parameters and *L* is the model’s likelihood given the data. When comparing models, the AIC weight of evidence for each model is given by $w\left(\mathrm{AIC}\right)\propto e^{‒\frac{1}{2}\Delta \left(\mathrm{AIC}\right)}$, where Δ(*AIC*) = *AIC* ‒ min(*AIC*) [29,30].

We also calculated Pearson’s correlation coefficients between evolutionary rates and the independent variable that defines each model. When comparing two models, we calculated partial correlation coefficients of evolutionary rates with the independent variable of each model controlling that of the other.

## Results and discussion

We aim to elucidate whether a site’s rate of evolution depends on flexibility or mutational stress as measured by MSF and MLmS, respectively. To address this issue, for each site of each of the 213 proteins of a dataset of monomeric enzymes, we used the Rate4Site program to estimate its relative evolutionary rate *ω*^*R*4*S*^, we calculated its *MSF* using both the pfANM model and the ANM model using Eq. (5), and we calculated its MLmS for the pfANM and ANM models using Eq. (15). We also considered as a measure of flexibility the B-factors of the pdb files. As described in Methods, all relative site-specific values were z-score normalized for each protein. All these values for the 77141 sites of the 213 proteins can be found in Additional file 2: Table S2.

### Stress vs. flexibility: protein-by-protein analysis

We started by performing a protein-by-protein comparison. For each protein, we fit the competing Stress and Flexibility models to the evolutionary rates *ω*^*R*4*S*^. Results for each protein can be found in Additional file 3: Table S3 and Additional file 4: Table S4. Summary information is shown in Table 1. The total AIC (summed over all proteins) is lower for the Stress Model than for the Flexibility model for either ENM potential. The mean AIC weight of evidence is much larger for the Stress Model than for the Flexibility Model. Moreover, mean AIC weights are consistent with counting the number of proteins for which one model outperforms the other: for the pfANM case, the Stress Model is best for 206/213 proteins and for the ANM case for 209/213 proteins. The (absolute value of the) average Pearson correlation is larger for MLmS than for MSF for both ENMs. Moreover, for both ENMs, while the mean partial rate-stress correlations are large, the mean partial rate-flexibility correlations, controlling for stress, are very small. In other words, MSF makes very little independent contribution to the explained variance of site-specific evolutionary rates.

**Table 1:** Model comparison: protein-by-protein analysis

**Potential**	**Model**	***y***	***x***	***AIC***	***<w*** **( *AIC *)** ***>***	***N***_***prot***_	***<R>***	***<pR>***
pfANM	Stress	*ω*	*MLmS*	190508	0.97	206	-0.54	-0.33
	Flexibility	*ω*	*MSF*	198662	0.03	7	0.45	-0.06
ANM	Stress	*ω*	*MLmS*	194154	0.98	209	-0.52	-0.39
	Flexibility	*ω*	*MSF*	207258	0.02	4	0.35	-0.04

NOTE: Potential is the ENM potential, Model is either the Stress Model or Flexibility Model, *y* is in all cases the site-specific rate of evolution inferred using Rate4Site, *x* is the independent variable of each model. *AIC* is the Akaike Information Criterion summed over all proteins. <*w(AIC)>* is the average of the AIC weight for each compared model (same ENM). ***N***_***prot***_ is the number of proteins for which each compared model (same ENM) is the best one. <*R>* is average over proteins of Pearson’s correlation coefficient between variables *y* and *x*. <*pR>* is the average partial correlation coefficient when controlling for the *x* variable of the contrasting model (same ENM). All variables were z-score normalized for each protein before fitting.

We think that it is most meaningful to compare between MLmS and MSF calculated using the same potential energy landscape (pfANM or ANM). However, the z-normalized MSF values can also be obtained from the B-factors available from the pdb files. We compared the Stress Model, both pfANM-based and ANM-based with a B-factor-based flexibility model and the conclusions are the same (results not shown). In general B-factor based Flexibility Models are the worst (see Additional file 3: Table S3 and Additional file 4: Table S4). This is not surprising because B-factors usually depend very strongly on several factors including experimental conditions, method used to estimate them, crystal disorder, etc. (see [31] and references therein).

To summarize, whether using the pfANM or the ANM potentials, stress (MLmS) predicts evolutionary rates better than dynamical flexibility (MSF) for almost all proteins of the dataset and the independent contribution of MSF is very small once MLmS is controlled for.

### Stress vs. flexibility: global analysis

To consider the total evidence in favour of each model we performed a global analysis. We obtained linear fits of the *ω*^*R*4*S*^ evolutionary rates to the Stress (Eq. 12) and Flexibility (Eq. 6) models for all 77141 sites of the dataset pooled together. Results are shown in Table 2. From AIC it follows that the Stress Model is better than the Flexibility Model for either pfANM or ANM. The Δ(*AIC*) values (within the same ENM) are so large that the total weight of evidence for the Stress Model, compared with the Flexibility Model, is *w*(*AIC*) = 1 whether using pfANM or ANM. The Pearson correlation coefficient R follows the same trend. We note, that even though smaller, the correlation coefficients for rate vs. MSF are significant, which agrees with previous findings [14,32,13,15]. However, partial correlations (pR) show that once stress (MLmS) is controlled for, the rate-MSF correlation almost disappears: the sequence-flexibility correlation is indirect. Similar results are obtained when using B-factors to estimate MSF (results not shown). In summary, the total evidence in support of the Stress Model is very strong.

**Table 2:** Model comparison: global analysis

**Potential**	**Model**	***y***	***x***	***AIC***	***w(AIC)***	***R***	***pR***
pfANM	Stress	*ω*	*MLmS*	191424	1.00	-0.55	-0.32
	Flexibility	*ω*	*MSF*	199645	0.00	0.47	-0.04
ANM	Stress	*ω*	*MLmS*	194589	1.00	-0.52	-0.40
	Flexibility	*ω*	*MSF*	207993	0.00	0.36	-0.02

NOTE: Results of global fits for all sites of the dataset. Potential is the ENM potential. Model is either the Stress Model or Flexibility Model. *y* is in all cases the site-specific rate of evolution inferred using Rate4Site, *x* is the independent variable of each model. *AIC* is the Akaike Information Criterion, that quantifies the goodness of fit of a model (the lower the better). *w(AIC)* is the AIC weight of evidence for each compared model (same ENM). *R* is Pearson’s correlation coefficient between variables *y* and *x. pR* is the partial correlation coefficient when controlling for the *x* variable of the contrasting model (same ENM). All variables were z-score normalized for each protein before fitting.

### Evolutionary rates vs. flexibility and stress

**Figure 1:**
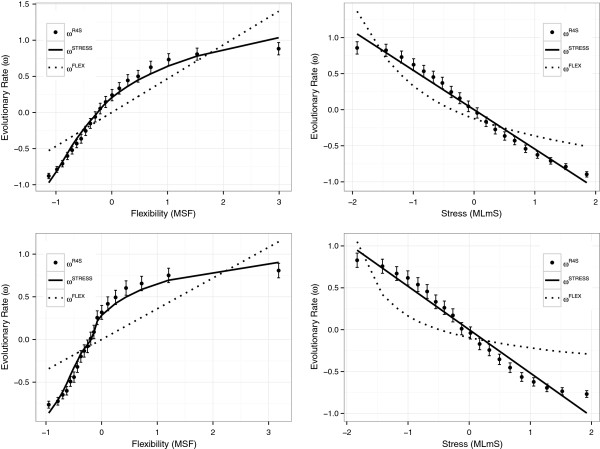
**Site-specific evolutionary rates vs. flexibility and stress.** Top panels: parameter-free Anisotropic Network Model (pfANM). Bottom panels: Anisotropic Network Model. Black circles: site-specific rates inferred from the sequence alignments using Rate4Site. Vertical segments: 99% confidence interval of the circles. Solid line: Stress Model predictions. Dashed line: Flexibility Model predictions. For the rate vs. flexibility plots (left panels), sites were evenly split between 20 bins according to their Mean Square Fluctuation (MSF) dynamical flexibility measure, and then MSF and evolutionary rates were averaged within bins. For the rate vs. stress plots (right panels), sites were evenly split into bins according to their Mean Local mutational Stress (MLmS), and average MLmS and rates were obtained for each bin. All variables were z-normalized for each protein.

What does the dependence of evolutionary rates on flexibility and stress look like? Figure 1 compares the inferred rates with the predictions of the Stress and Flexibility models. The models were globally fit as described in the previous section. Clearly, the Stress Model fits the inferred rates nicely over almost the whole range, in evident contrast with the Flexibility Model, for both pfANM (top panels) and ANM (bottom panels).

Even though previous sequence-flexibility studies used Pearson correlations, which, rigorously, make sense only for linear relationships, they already found nonlinear sequence-flexibility plots similar to those of Figure 1 (left panels) [14,32,13]. In spite of this, they either dismissed the nonlinear part [14]or interpreted it in terms of different selection regimes [13]. From Figure 1 (left panels) it is clear that the nonlinearity follows naturally from the proposed Stress Model, suggesting that evolutionary rates depend nonlinearly on *MSF* because they depend (approximately) linearly on *MLmS*, and *MSF* ≈ 1/*MLmS*, which can be derived from Eqs. (19) and (20).

To conclude this subsection, we must observe that inferred rates are larger than stress-based predictions for the slowest sites and smaller for the fastest. A reason could be that inference methods overestimate small rates and underestimate large ones [33]. However, close inspection of the rate vs. stress curves (right panels of Figure 1) indicates that despite the very good fit of the linear Stress Model, there still seems to be some remaining nonlinearity of the *ω*^*R*4*S*^ vs. *MLmS* plots. A possible reason is the weak-selection approximation used to linearize the exponential in Eq. (11), however, resolving this issue is beyond the scope of the present report.

### pfANM vs. ANM

To finish this section, we compare ANM with pfANM. Figure 1 shows that both pfANM and ANM result in similar qualitative dependence of rate vs. flexibility (left panels) and rate vs. stress (right panels). However, the pfANM potential (top panels) results in better fits to the inferred rates than the ANM potential (bottom panels). Accordingly, the AIC values (Table 1 and Table 2) show that the pfANM-based stress model is better than the one based on ANM. This is in agreement with the finding that WCN correlates better than CN with evolutionary rates [11].

## Conclusion

We introduced a mechanistic Stress Model of protein sequence evolution. Mutations are modelled as random perturbations of the protein’s potential energy landscape, represented using Elastic Network Models. To model natural selection, we used basic statistical physics to derive the expected probability that a mutant samples a specific functional structure. From this, we deduced a linear relationship between a site’s mean evolutionary rate and the mean local mutational stress (MLmS) of the functional conformation. We compared this model with an empirical Flexibility Model that postulates that a site’s evolutionary rate is linearly dependent on its flexibility (measured by its MSF). We compared both models and found strong support for the Stress Model. Moreover, the independent contribution of flexibility is negligible once stress is controlled for.

The MLmS is proportional to Local Packing Density and, therefore, the Stress Model provides a mechanism for the connection between a site’s LPD and its evolutionary rate. Regarding the sequence-flexibility relationship, previous empirical correlation studies had already found that the sequence-flexibility relationship is nonlinear and either dismissed the nonlinear parts or attempted an interpretation in terms of different selection regimes [14,32,13]. We found the nonlinearity follows naturally from the Stress Model: evolutionary rates depend nonlinearly on *MSF* because they depend (approximately) linearly on *MLmS*, and *MSF* ≈ 1/*MLmS*. To summarize, the Stress Model accounts for the observed site-dependency of evolutionary rates and its relationship with packing density and flexibility.

A note of caution is in order here. For the Stress Model mutational stress was not postulated to be the determinant factor *a priori* but, rather, it was derived from the assumptions of the model that are essentially two (1) there is a single active conformation and (2) mutants are flexible and therefore can sample the active conformation so that they are at least partly functional. Therefore even though Stress Model was chosen to designate this mechanistic model, it should be kept in mind that it demonstrates the importance of protein flexibility.

It is worthwhile to mention some of the possible caveats and further developments of the Stress Model. First, we assume a single active conformation. In principle, it would be reasonable to assume that only changes of the active-site conformation should affect fitness. However, we note that if protein sites are strongly coupled, which is often the case, any conformational change will affect the active site conformation. For a strongly coupled elastic network forcing the active site to adopt a given conformation makes the rest of the protein move accordingly. Therefore, assuming that the whole protein conformation must be in the “active conformation” for the protein to function is not necessarily an important limitation. However, for cases where the coupling is not very strong, if the active site is known, this could be easily tackled using a modified version of the selection function that integrates away all coordinates except for those of the active site (i.e. uses marginal conformational distributions rather than the full ones in the definition of selection function).

Second, in Eq. (11) we performed a linear approximation of the exponential function. This is reasonable *a priori* only for weak selection, and *a posteriori* by the good fit of the resulting model to the data. We should note, however, this approximation can be easily removed, and the actual mean of the exponential can be calculated via simulation. Further work is needed to explore this possibility.

Third, we note that the z-normalized MLmS values, on which the Stress Model is based, are identical to the z-normalized LPD measures WCN (for the pfANM potential) and CN (for the ANM potential). For other potentials this need not be the case and it is for that reason that we chose to keep the notation MLmS in the present tables and figures, to make them comparable with further research based on estimating MLmS using different, perhaps better, potential energy functions.

To close, we note that the mutational part of the Stress Model accounts for observed patterns of evolutionary divergence of protein structure and dynamics [21-23]. Regarding structural divergence, unselected random mutations reproduce very well the evolutionary conservation of a “structural core” and account for the observation that structures diverge mainly within the space spanned by a few low-energy collective normal modes [21,22]. Regarding protein motions, unselected random mutations explain the higher conservation of the low-energy normal modes in terms of their mutational robustness [31,23]. In general, those studies could found no evidence of natural selection at the levels of structural or dynamical divergence. Clearly, without natural selection, all sites would evolve at the same rate, which is not the case. The Stress Model proposed here accounts rather well for the variation of rates of evolution among sites. It would be interesting to study the effect of the selection function introduced here on structural and dynamical divergence and compare the observed patterns with those that result from unselected mutations. This could advance our understanding of the effect of selection at the levels of structure and dynamics. In general, we think the Stress Model provides a possible unifying framework to study evolutionary protein divergence at the levels of sequence, structure, and dynamics.

## Abbreviations

RSA: Relative solvent accessibility; LPD: Local packing density; WCN: Weighted contact number; CN: Contact number; ENM: Elastic network model; ANM: Anisotropic network model; pfANM: parameter-free anisotropic network model; MSF: Mean square fluctuation; MLmS: Mean local mutational stress; AIC: Akaike information criterion.

## Competing interests

The authors declare that they have no competing interests.

## Authors’ contributions

JE and JKH designed the study. JE derived the Stress Model. TTH and MLVM wrote the codes and performed the calculations and analysed data. JE drafted the manuscript. All authors commented on and approved the final manuscript.

## Supplementary Material

Additional file 1: Table S1 Protein Dataset. Protein: pdb code; chain: protein chain; EC.class: Enzyme Comission class; SCOP class: structural class of domains according to the SCOP classification; Domains: number of domains; Year: year in which the structure was determined; Resolution: resolution of the X-ray experiment; Sites: number of sites of the protein chain; Number.of.Sequences: number of sequences of the multiple sequence alignment used for site-specific rate inferences. Click here for file

Additional file 2: Table S2 Site-specific rates, flexibility, and stress measures. pdb: pdb identifier of the protein; chain: protein chain; site: protein site; zwr4s: z-normalized site-specific rate of evolution inferred using Rate4Site; zbfactor: z-normalized B-factor; zmsf_pfanm: z-normalized Mean Square Fluctuation (MSF) obtained with the pfANM model; zmlms_pfanm: z-normalized Mean Local mutational Stress (MLmS) obtained with the pfANM model; zmsf_anm: z-normalized MSF for the ANM model; zmlms_anm: z-normalized MLmS for the ANM model. Click here for file

Additional file 3: Table S3. Protein by protein goodness-of-fit measures. pdb: pdb identifier of the protein; chain: protein chain; aic.zmlms_pfanm: Akaike Information Criterion (AIC) of the pfANM-based Stress Model; aic.zmsf_pfanm: AIC of the pfANM-based Flexibility Model; aic.zmlms_anm: AIC for the ANM-based Stress Model; aic.zmsf_anm: AIC for the ANM-based Flexibility Model: AIC.zbfactor: AIC for a B-factor based Flexibility Model; r.zmlms_pfanm: Pearson correlation coefficient (R) of the pfANM-based Stress Model; r.zmsf_pfanm: R of the pfANM-based Flexibility Model; r.zmlms_anm: R for the ANM-based Stress Model; r.zmsf_anm: R for the ANM-based Flexibility Model; r.zbfactor: R for a B-factor-based Flexibility Model. Click here for file

Additional file 4: Table S4 Protein by protein comparison of pairs of models. pdb: pdb identifier of the protein; chain: protein chain; waic.m1.m2 is the Akaike Information Criterion weight of evidence w(AIC) of m1 in an m1 vs m2 comparison. pr.m1.m2 is the partial correlation of site-specific rates of evolution with the independent variable defining m1, controlling that of m2. Models considers are: pfANM-based Stress Model (zmlms_pfanm); pfANM-based Flexibility Model (zmsf_pfanm); ANM-based Stress Model (zmlms_anm); ANM-based Flexibility Model (zmsf_anm); B-factor-based Flexibility Model (zbfactor). Click here for file
